# Glia from the central and peripheral nervous system are differentially affected by paclitaxel chemotherapy *via* modulating their neuroinflammatory and neuroregenerative properties

**DOI:** 10.3389/fphar.2022.1038285

**Published:** 2022-11-02

**Authors:** Ines Klein, Janne Boenert, Felix Lange, Britt Christensen, Meike K. Wassermann, Martin H. J. Wiesen, Daniel Navin Olschewski, Monika Rabenstein, Carsten Müller, Helmar C. Lehmann, Gereon Rudolf Fink, Michael Schroeter, Maria Adele Rueger, Sabine Ulrike Vay

**Affiliations:** ^1^ University of Cologne, Faculty of Medicine and University Hospital Cologne, Department of Neurology, Cologne, Germany; ^2^ Center of Pharmacology, Therapeutic Drug Monitoring, University Hospital of Cologne, Cologne, Germany; ^3^ Cognitive Neuroscience, Institute of Neuroscience and Medicine (INM-3), Research Centre Juelich, Juelich, Germany

**Keywords:** paclitaxel, chemotherapy-related neurotoxicity, neuroinflammation, astrocytes, microglia, satellite glia cells, neural stem cells, BDNF (brain derived neurotrophic factor)

## Abstract

Glia are critical players in defining synaptic contacts and maintaining neuronal homeostasis. Both astrocytes as glia of the central nervous system (CNS), as well as satellite glial cells (SGC) as glia of the peripheral nervous system (PNS), intimately interact with microglia, especially under pathological conditions when glia regulate degenerative as well as regenerative processes. The chemotherapeutic agent paclitaxel evokes peripheral neuropathy and cognitive deficits; however, the mechanisms underlying these diverse clinical side effects are unclear. We aimed to elucidate the direct effects of paclitaxel on the function of astrocytes, microglia, and SGCs, and their glia-glia and neuronal-glia interactions. After intravenous application, paclitaxel was present in the dorsal root ganglia of the PNS and the CNS of rodents. *In vitro*, SGC enhanced the expression of pro-inflammatory factors and reduced the expression of neurotrophic factor NT-3 upon exposure to paclitaxel, resulting in predominantly neurotoxic effects. Likewise, paclitaxel induced a switch towards a pro-inflammatory phenotype in microglia, exerting neurotoxicity. In contrast, astrocytes expressed neuroprotective markers and increasingly expressed S100A10 after paclitaxel exposure. Astrocytes, and to a lesser extent SGCs, had regulatory effects on microglia independent of paclitaxel exposure. Data suggest that paclitaxel differentially modulates glia cells regarding their (neuro-) inflammatory and (neuro-) regenerative properties and also affects their interaction. By elucidating those processes, our data contribute to the understanding of the mechanistic pathways of paclitaxel-induced side effects in CNS and PNS.

## Graphical Abstract

**Figure:**
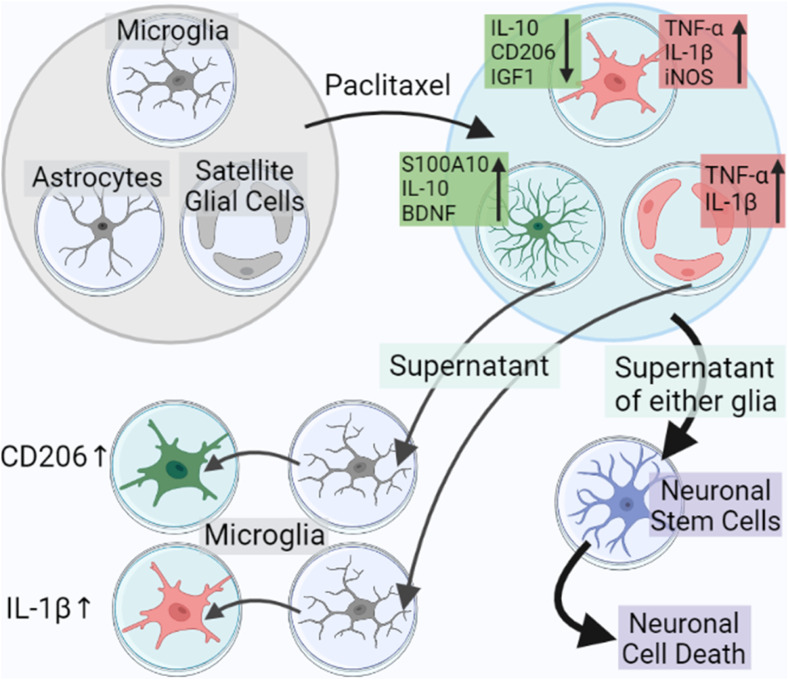
Main Points: Paclitaxel differentially modulates inflammatory and regenerative properties of glial cells; Paclitaxel affects glia-glia and glia-neuron interactions; Paclitaxel induces pro-inflammatory effects in microglia and satellite glial cells and anti-inflammatory effects in astrocytes.

## 1 Introduction

Paclitaxel is a widely used effective anti-neoplastic agent applied to treat various cancer entities. One of the significant side effects of paclitaxel is chemotherapy-induced peripheral neuropathy (CIPN). CIPN is associated with length-dependent axonal sensory neuropathy, and symptoms like numbness, tingling, or allodynia, develop in up to 97% of all treated patients (Tanabe et al., 2013; Staff et al., 2017; AkhileshUniyal et al., 2022). The main effects of paclitaxel are symmetrical neuronal damage and die-back of peripheral axons (Argyriou et al., 2008; Carlson and Ocean, 2011). In the PNS, microglia recruitment into the DRG (Chen et al., 2017) and SGC activation were described after paclitaxel treatment (Warwick and Hanani, 2013). Furthermore, activation of astrocytes and microglia was reported in the spinal cord, contributing to the pathogenesis of paclitaxel-induced peripheral neuropathy (Liu et al., 2019; Pevida et al., 2013; Zhang et al., 2012). This activation included increased expression levels of TNF-α (Bogdan and Ding, 1992), IL-1β (Voloshin et al., 2015), IL-6 (Huehnchen et al., 2020) and IL-10 (Ledeboer et al., 2007).

Paclitaxel crosses the blood-brain barrier and accumulates in the hippocampus and cortex, as previously demonstrated in rodents (Huehnchen et al., 2017). CNS-effects of paclitaxel were suggested to be associated with impaired adult hippocampal neurogenesis (Ahles and Saykin, 2007). Besides, several studies reported chemotherapy-induced CNS neurotoxicity (Joly et al., 2015). In humans, paclitaxel-induced CNS side effects include a cognitive decline in verbal and visuospatial abilities, executive functions, processing speed, and attention span (Joly et al., 2015). To date, the main focus of paclitaxel-induced peripheral neuropathy refers to neuronal damage in the CNS and PNS, respectively. The effects of paclitaxel on glial cells have so far not been described accurately. Glia constitute a significant fraction of the CNS and the PNS tissue. They take over supportive functions for the neurons, defining synaptic contacts and maintaining neuronal signals. Microglia exist ubiquitously in the CNS and the PNS. They are resident immune-competent cells and regularly screen the microenvironment, maintain cell homeostasis, and interact with neuronal tissue *via* overly motile processes and protrusions (Nimmerjahn et al., 2005). Under physiological conditions, microglia are critical players in neuronal development, function, and synaptic plasticity (Ekdahl, 2012; Kettenmann et al., 2013; Sierra et al., 2014a; Sierra et al., 2014b). With changes in brain homeostasis or due to neurological diseases, they become activated and release cytokines, chemokines, and growth factors, fostering either neurotoxic or neuroprotective effects (Butovsky et al., 2014; Tang and Le, 2016). They phagocyte and interact with infiltrating immune cells. There are multiple microglia phenotypes, with the most described being the “classically”-activated (M1) microglia secreting pro-inflammatory cytokines, and nitric oxide (NO), inducing neuronal loss and impending tissue repair. Microglia that release neurotrophic factors and promote healing and repair are often termed “alternatively”-activated (M2) (Franco and Fernández-Suárez, 2015; Tang and Le, 2016). Astrocytes are supportive glial cells in the CNS that supply the brain with nutrients, maintain extracellular ion balance, and take over key roles in the brain’s and spinal cord’s repair processes. This “reactive astrogliosis” process is one of the hallmarks of diseased CNS tissue. Further, they support endothelial cells, thereby (co-) regulating cerebral blood flow and the blood-brain barrier (Freeman and Rowitch, 2013). Recently, differential activation states were described for astrocytes, similar to those for microglia. Within these phenotypes, pro-inflammatory astrocytes (A1) have been described to upregulate classical complement cascade genes potentially destructive to synapses. In contrast, anti-inflammatory astrocytes (A2) increase neurotrophic factors’ expression, hence exerting neuroprotective properties (Liddelow et al., 2017). Satellite glial cells (SGCs) are supportive glial cells of the PNS enveloping neurons in the dorsal root ganglia (DRG). Under pathological circumstances, they upregulate glial fibrillary acidic protein (GFAP), strengthen intercellular communication *via* upregulation of Connexin 43 (CX43), and increase cytokine expression (Hanani and Spray, 2020). SGCs regulate neuronal activity and pain sensation (Kim et al., 2016; Komiya et al., 2018).

Since glia are central regulators of neuronal function, activity, and survival, we hypothesized that paclitaxel might indirectly exert toxic effects on neurons by affecting glial functions in the CNS and PNS. Due to their similarities in morphology and function, we assumed that astrocytes of the CNS and satellite glial cells of the PNS react similarly to the same stimulus. To investigate this issue, we first confirmed the presence of paclitaxel in the CNS after intravenous injection of paclitaxel in mice. Furthermore, we treated primary astrocyte, microglia or SGC cultures with paclitaxel and investigated their morphology, activation state and (inflammatory) expression profile *in vitro*. We then investigated the neurotoxic effect of the secretome of paclitaxel-treated glia cells on differentiated neural stem cells.

## 2 Materials and methods

### 2.1 Animals

Animal experiments and animal procedures for tissue harvesting obeyed the German Federal Laws for Animal Protection and were approved by the local state authorities (Landesamt für Natur, Umwelt und Verbraucherschutz Nordrhein-Westfalen; LANUV NRW AZ 84-02.04.2012.A116, and the local animal care committee, AZ UniKöln_Anzeige §4.16.021 and AZ UniKöln_Anzeige §4.21.005). Animals were maintained in polysulfone cages with sawdust bedding on a 12 h light/dark cycle. Food and water were provided *ad libitum* in pathogen-free conditions. Animal species, line, age, and gender are described in more detail in the respective subheaders. Twelve male C57BL/6 mice were used for the *in vivo* experiments, and for the *in vitro* experiments, a total of 44 (25 female, 19 male) neonatal Wistar rats derived from four different litters were used.

### 2.2 Liquid chromatography-tandem mass spectrometry (LC-MS/MS)

Twelve 8-week-old male C57BL/6 mice (Jackson Laboratory) were used to measure paclitaxel amounts in the brain. Animals were injected intravenously with 25 mg/kg paclitaxel (obtained from the University Hospital Cologne, Germany) before they were deeply anaesthetized with 6.5 μL/g body weight of 120 mg/kg ketamine (INRESA Arzneimittel GmbH, Germany) and 20 mg/kg xylazine (Bayer AG, Germany). Serum was collected 0.5 h, 1.5 h, and 3 h after paclitaxel injection, and subsequently, animals were euthanized *via* perfusion with 1x phosphate-buffered saline (PBS; Thermo Fisher Scientific, United States) or cervical dislocated 0.5 h, 1.5 h, and 3 h after paclitaxel injection. After removing the forebrain, hippocampus and cortex were dissected and homogenized with H_2_O (2.5 g brain tissue/100ml; Thermo Fisher Scientific, United States). 50 μl homogenate or serum were mixed with 50 μl of extraction solution acetonitrile (Sigma-Aldrich, United States) and 50 μl of internal standard solution prepared with acetonitrile ([13C6]-paclitaxel obtained from Alsachim (Illkirch Graffenstaden, France)). Samples were thoroughly mixed and centrifuged at 12,000 rpm for 10 min. The clear supernatants were transferred into LC-MS/MS glass vials and supplied into the LC-MS/MS system. LC-MS/MS protocol was followed, and paclitaxel quantification was carried out as described previously (Klein et al., 2021).

### 2.3 Satellite glial cell culture

Ten male and female neonatal Wistar rats (P1-P3) were euthanized *via* decapitation to generate SGC cultures from dorsal root ganglia (DRG). Excess skin and tissue were removed to access the spine. The spine was longitudinally cut in half, and DRGs were removed and placed in 2 ml Leibovitz’s L-15 Medium (Thermo Fisher Scientific, United States). DRGs were digested with trypsin/ethylenediaminetetraacetic acid (EDTA) solution (0.05% trypsin, 0.02% EDTA; PAN-Biotech GmbH, Germany) for 15 min at 37°C and afterwards dissociated by trituration. DRG homogenate of two animals was pooled and centrifuged at 1,200 rpm for 5 min, and the resulting pellet was, after assessing cell numbers, seeded into cell culture flasks accordingly. Cells were cultured in Dulbecco’s essential medium (DMEM; Thermo Fisher Scientific, United States) with 10% fetal bovine serum (FBS; PAN-Biotech GmbH, Germany), 1% penicillin/streptomycin (PAN-Biotech GmbH, Germany), and 2 mM L-glutamine (Thermo Fisher Scientific, United States). Cells were maintained at 37°C with 5% CO_2_ for 7 days. For experiments, SGC were detached using trypsin/EDTA solution (0.05% trypsin, 0.02% EDTA) for 15 min at 37°C. The trypsin/cell suspension was centrifuged for 2 min at 1,200 rpm, the supernatant was removed, and the SGCs were resuspended in fresh culture medium and seeded for experiments. The protocol was adapted as previously described (Wang et al., 2019). Purity of glial cell culture was verified *via* Glial Fibrillary Acidic Protein Antibody, clone GA5 (GFAP; 1:500; Millipore Cat# MAB360, RRID:AB_11212597), CX43 (Sigma-Aldrich Cat# C6219, RRID:AB_476857), CNPase (Merck Millipore Cat# MAB326, RRID:AB_2082608) and MBP1 (Abcam Cat# ab40390, RRID:AB_1141521) staining (data not shown).

### 2.4 Astrocyte and microglia isolation and culture

Pure neonatal astrocytes and microglia cultures were obtained from the cortices of 20 male and female neonatal Wistar rats (P1-P3) according to the protocol of Vay et al., 2021. Rat cortices were incubated in trypsin/EDTA solution (0.05% trypsin, 0.02% EDTA) for 15 min at 37°C. The addition of the culture medium (DMEM with 10% FBS, 1% penicillin/streptomycin, and 2 mM L-glutamine) stopped the reaction. The cortices were dissociated by repeated up- and down-pipetting. Four cortices cell suspensions were pooled and centrifuged at 1,200 rpm for 2 min. Cells were resuspended in DMEM (10% FBS, 1% penicillin/streptomycin, 2 mM L-glutamine) and grown at 37°C with 5% CO2 for 8–10 days. The culture medium was changed after the third day. This prolonged co-cultivation approach promoted a selective growth of astrocytes and microglia. Microglia were then obtained as previously described (Rabenstein et al., 2016). Culture-flasks were shaken at least three times for 1 h at 250 rpm on an orbital shaker (37°C) to separate microglia from astrocytes. The medium containing detached microglia was centrifuged at 1,200 rpm for 3 min and resulting microglia were resuspended in fresh medium and subcultured in 24-well-plates at 50,000/well for the experiments. The remaining astrocytes in the flasks were detached using trypsin/EDTA solution (0.05% trypsin, 0.02% EDTA) for 15 min at 37°C. The trypsin/cell suspension was centrifuged for 2 min at 1,200 rpm and the supernatant was removed. The obtained pure astrocyte pellet was resuspended in fresh culture medium and seeded for experiments.

### 2.5 Neural stem cell isolation, cultivation, and differentiation

Neuronal stem cells (NSCs) from fetal Wistar rat cortices were derived from embryonic day 13.5 following previously published protocols (Rueger et al., 2010; Vay et al., 2016). Fetal cortices were dissociated in DMEM/F12 medium (Thermo Fisher Scientific, United States) plus 1% N2 supplement (Thermo Fisher Scientific, United States), 1% penicillin/streptomycin, 0.6 mM L-glutamine, and 1% sodium pyruvate (Thermo Fisher Scientific, United States) by repeated up- and down-pipetting and the resulting cell suspension was sown on 10 cm petri dishes for expansion. To obtain monolayer cultures, dishes were pre-coated with L-poly-ornithine (15%; Sigma-Aldrich, United States) and bovine fibronectin (2.5 mmol/L; Sigma-Aldrich, United States). NSC were grown at 37°C with 5% CO2 and human recombinant basic Fibroblast Growth Factor (FGF-2; 10 ng/ml; Sigma-Aldrich, United States) was included throughout culturing and medium was changed every second day. For differentiation, cells from the second or third passage were sown on pre-coated coverslips in 24-well plates at 20,000/well in the presence of FGF-2. After 24 h, differentiation was initiated by withdrawing the mitogen FGF-2. By differentiation of primary NSC for 7 days, robust young neurons, astrocytes, a few oligodendrocytes, and residual undifferentiated NSCs are present in the differentiating cultures (Vay et al., 2016; Vay et al., 2018).

### 2.6 Cell treatment

#### 2.6.1 Microglia, astrocytes, and satellite glial cells treatment

Microglia, astrocytes and SGC were separately cultured in 24-well plates at 50,000/well in DMEM (10% FBS, 1% penicillin/streptomycin, 2 mM L-glutamine). Cells were cultured in white 96-well plates at a density of 12,000/well. After 24 h, cells were either not treated (control) or treated with 1 μM, 5 μM, 10 μM or 50 μM paclitaxel (obtained from University Hospital Cologne, Pharmacy) for 24 h before cell viability assay, NO- or ATP-assay, Enzyme-linked immunosorbent assay (ELISA), or RNA-extraction were performed (Lehmann et al., 2020). For immunocytochemical staining, cells were fixed with 4% paraformaldehyde (PFA; Carl Roth, Germany).

To obtain “conditioned medium” microglia, astrocytes and SGCs were either not stimulated (conditioned medium-control) or stimulated with paclitaxel 1 μM, 5 μM, 10 μM, or 50 μM for 24 h. After washing cells with 1x PBS, cells were incubated with fresh paclitaxel-free cell culture medium (DMEM containing 10% FBS, 1% penicillin/streptomycin, 2 mM L-glutamine) for 24 h. Conditioned medium containing the secretome but no paclitaxel was collected and stored at −20°C.

#### 2.6.2 Neuronal stem cell treatment

After 7 days of differentiation, when neurons and glia cells were formed robustly, NSCs were treated with the conditioned medium obtained from paclitaxel-stimulated cultures of astrocytes, microglia, and SGCs (cmp. “cell treatment”). NSC treated with purely “glial cell culture medium” DMEM containing 10% FBS, 1% penicillin/streptomycin, and 2 mM L-glutamine served as control. After 24 h, cell viability assay was conducted, or NSCs were fixed with 4% PFA and immunocytochemically stained to visualize young neurons and astrocytes (see immunohistochemistry *in vitro*).

### 2.7 Cell viability assay

After 24 h exposure to 1 μM, 5 μM, 10 μM, and 50 μM paclitaxel apoptotic microglia, astrocytes and SGCs, as well as neuronal stem cells after exposure to glial supernatant, were stained with propidium iodide (Life Technologies, Darmstadt, Germany) and all cells were counterstained with Hoechst 33342 (Life Technologies, Darmstadt, Germany) following the protocol as previously described (Vay et al., 2021). Images were taken with the BZ-9000 microscope (Keyence, Japan). Ten images per well were taken, and Hoechst- and propidium iodide-stained cells were counted manually. The ratio of propidium iodide positive on total cell count provided the proportion of cell death. The experiment was performed in triplicate with three wells per condition. The mean values ± standard error of the mean (SEM) were established among equally treated samples.

### 2.8 ATP assay

Following the manufacturer’s protocol, ATP amounts after exposure to 1 μM and 5 μM paclitaxel were detected in microglia, astrocytes and SGCs using the Luminescent ATP Detection Assay Kit (ab113849, Abcam, United States) following the manufacturer’s protocol. Luminescence was measured using a plate reader (FLUOstar Omega, BMG LABTECH, Germany). Each experiment was conducted in triplicate with at least 10 wells per condition. Mean values ± SEM were established among equally treated samples.

### 2.9 Griess assay

Released nitric oxide (NO) was measured in the cell culture medium of cultured and treated (1 μM and 5 μM paclitaxel) microglia, astrocytes and SGCs using the Griess reagent kit (#30100, Biotium, United States). According to the suppliers' protocol, the nitric oxide (NO) amount was detected photometrically at 548 nm in a plate reader (FLUOstar Omega, BMG LABTECH, Germany). Mean values ± SEM were established among equally treated samples. Each experiment was conducted in triplicate.

### 2.10 Real-time quantitative PCR

RNA from cultured microglia, astrocytes and SGCs was collected using the MasterPure Complete DNA & RNA Purification Kit (MC85200, Lucigen, United Kingdom) after exposure to control medium, and 1 μM and 5 μM paclitaxel. The manufacturer’s protocol was followed, and total RNA and purity were photometrically measured at 260 nm. cDNA was synthesized using the QuantiTect Reverse Transcription Kit (#205311, Qiagen, Germany) following the supplier’s instructions. All primers were purchased from Biolegio (Nijmegen, Netherlands) and diluted to a final concentration of 10 pmol/μl. Primer sequences and PCR parameters are listed in Table 1. RT-qPCR was performed on a Bio-Rad CFX ConnectTM real-time system (Hercules, United States) or LightCycler 96 (Roche, Germany). PCR product integrity was evaluated by melting point analysis and agarose gel electrophoresis. The obtained threshold cycle (CT) was normalized to housekeeping gene 60s ribosomal protein L13a (RPL13a; ΔCT), and the result was normalized to the control condition (ΔΔCT). Experiments were performed in technical triplets, and means were depicted as 2^(−ΔΔCT)^. Mean values were calculated for all samples. Each experiment was conducted in at least triplicate.

**TABLE 1:** Used primers and parameters of RT-qPCR.

RNA	Sequences forward/reverse 5′–3′	Temperature (°C) step 1/2/3	Duration (s) step 1/2/3	Accession number
BDNF	TAC​CTG​GAT​GCC​GCA​AAC​AT/TGG​CCT​TTT​GAT​ACC​GGG​AC	94/60/72	15/30/45	M61175
Complement C3 (C3)	ATC​GAG​GAT​GGT​TCA​GGG​GA/GCC​TCT​ACC​ATG​TCG​CTA​CC	95/60/72	15/15/15	NM_016994.2
CD206	AAC​AAG​AAT​GGT​GGG​CAG​TC/CCT​TTC​AGT​CCT​TTG​CAA​GC	95/56/72	15/15/45	NM_001106123.2
Connexin 43 (CX43)	CTT​GGA​GCC​GTG​TAG​CTG​TG/GTA​GCA​ATG​GCA​TGG​TTG​GTG	95/60/72	15/15/45	NM_012567.2
GFAP	TGC​ATG​TAC​GGA​GTA​TCG​CC/GGG​GGA​GGA​AAG​GAC​AAC​TG	95/60/72	15/15/15	NM_017009.2
IL-10	TTT​CCA​AGG​AGT​TGC​TCC​CG/GAA​AAA​TTG​AAC​CAC​CCG​GCA	95/56/72	15/15/45	NM_012854.2
IL-1β	GAC​TTC​ACC​ATG​GAA​CCC​GT/GGA​GAC​TGC​CCA​TTC​TCG​AC	95/56/72	15/15/45	NM_031512.2
IL-6	CCC​AAC​TTC​CAA​TGC​TCT​CCT/AGC​ACA​CTA​GGT​TTG​CCG​AG	95/57/72	15/15/45	NM_012589.2
iNOS	GCT​TGT​CTC​TGG​GTC​CTC​TG/CTC​ACT​GGG​ACA​GCA​CAG​AA	95/59/72	15/15/45	NM_012611.3
Ki67	TCT​TGG​CAC​TCA​CAG​TCC​AG/GCT​GGA​AGC​AAG​TGA​AGT​CC	95/58/72	15/15/45	NM_001271366.1
NT-3	CAG​CCC​TTT​TGA​GGG​ACC​AT/GGA​TGC​CAC​GGA​GAT​AAG​CA	95/60/72	15/15/45	NM_001270870.1
RPL13a	TCT​CCG​AAA​GCG​GAT​GAA​CAC/CAA​CAC​CTT​GAG​GCG​TTC​CA			NM_173340.2
S100A10	CAC​ACC​TTG​ATG​CGT​CCT​CT/GGC​AAC​CGG​ATG​CAA​ACA​AT	95/59/72	15/15/45	NM_031114.1
TNF-α	CAT​CCG​TTC​TCT​ACC​CAG​CC/AAT​TCT​GAG​CCC​GGA​GTT​GG	95/57/72	15/15/45	NM_012675.3
Vimentin	GCA​GCC​TCT​ATT​CCT​CGT​CC/TAG​TTG​GCG​AAG​CGG​TCA​TT	95/60/72	15/15/15	NM_031140.1

### 2.11 Enzyme-linked immunosorbent assays

Concentrations of tumour necrosis factor-α (TNF-α), interleukin 1β (IL-1β), interleukin 6 (IL-6), as well as insulin-like growth factor 1 (IGF1) were measured in the cell culture medium of treated microglia, astrocytes and SGCs using rat TNF-α, IL-1β or IL-6 DuoSet ELISA Kit (Cat# DY510-05, #DY501, #DY506; R&D systems, United States), and the mouse/rat IGF1 Quantikine ELISA Kit (Cat# MG100, R&D systems, United States). The manufacturer’s protocol was followed, and samples were measured using a plate reader (FLUOstar Omega, BMG LABTECH, Germany). Product concentrations were calculated based on standard curves. Mean values ± SEM were established among equally treated samples. Each experiment was conducted in at least triplicate.

### 2.12 Bromodeoxyuridine proliferation assay

Bromodeoxyuridine (BrdU; Cayman Chemical Company, United States) is a thymidine analogue and serves to label and quantify proliferating cells. Eighteen hours after treatment of microglia, astrocytes and SGCs with 1 μM or 5 μM paclitaxel, 10 µM BrdU was offered to each well as previously described (Vay et al., 2021). After 6 h of incubation, the experiment was stopped by cell fixation with 4% PFA, and cells were stained for incorporated BrdU (see below). Only cells that underwent the S-phase of the cell cycle have incorporated BrdU and represent fluorescent staining of the cell nucleus. Ten pictures of each sample were taken using an inverted fluorescence phase-contrast microscope, and at least 250 total cells per sample and experiment were counted manually. The ratio of BrdU-positive cells on total cell count provided the proportion of proliferating cells. The experiment was performed in triplicates with four wells per condition. The resulting mean values ±SEM were established among equally treated samples.

### 2.13 Immunohistochemistry *in vivo*

For representative images of paclitaxel influx into CNS and PNS tissue, six 8-week-old male C57BL/6 mice (Jackson Laboratory) were used. Animals were injected intravenously with 25 mg/kg paclitaxel (obtained from University Hospital Cologne, Germany) before they were deeply anesthetized with 6.5 μL/g body weight of 120 mg/kg ketamine and 20 mg/kg xylazine. Mice were perfused with 1x PBS and euthanized *via* cervical dislocation 0.5 h after paclitaxel injection. The brain and DRGs were collected and subsequently used for immunohistochemistry as previously described (Klein et al., 2021). Brain and DRGs were fixed with 4% PFA, and after cryoprotection with 30% sucrose (Sigma-Aldrich, United States) tissue was cut on a cryostat (30 µm sections). Tissue was stained with primary 1:500 anti-Taxol IgG (Abcam Cat# ab26953, RRID:AB_778261) for 1 h at room temperature and secondary antibody AlexaFluor488 (1:500) for 1.5 h at room temperature. For visualization of cell nuclei, Hoechst 33342 (1:200) was used. A × 20/0.75 numerical aperture objective lens on the BZ-9000 microscope (Keyence) was used to examine stained samples.

### 2.14 Immunohistochemistry *in vitro*

Cells were fixed with 4% PFA for 10 min. Staining against GFAP (1:500; Millipore Cat# MAB360, RRID:AB_11212597) was used to identify astrocytes. Marking the cytoskeleton of cells by anti-vimentin, clone V9 (1:250; Millipore Cat# MAB3400, RRID:AB_94843) presents the shape of cells. The purity of astrocytes cultures was regularly verified by conducting co-staining with anti-GFAP and anti-ionized calcium-binding adapter molecule 1 (Iba1; 1:500; Abcam Cat# ab5076, RRID:AB_2224402) to detect microglia. The maximal fraction of microglia was <10%. To identify the astrocyte phenotype, anti-complement component 3, clone 12E2 (C3; 1:500; Thermo Fisher Scientific Cat# HYB 118-02-02, RRID:AB_2066731) and anti-S100A10 (1:50; Novus Cat# NBP1-89370, RRID:AB_11012229) were used. Furthermore, the translocation of resting nuclear factor kappa-light-chain-enhancer of activated B cells (NFκB) from the cytoplasm into the cell nuclei of reactivated astrocytes was identified using anti-NFκB p65 (1:500; Thermo Fisher Scientific Cat# PA5-16545, RRID:AB_10981081). Hemichannels of astrocytes are mainly composed of Connexin 43 (CX43) protein, which was detected using anti-CX43 (1:8,000; Sigma-Aldrich Cat# C6219, RRID:AB_476857). To assess the proliferation rate *via* BrdU-incorporation, astrocytes were stained with anti-BrdU (1:200; Sigma-Aldrich Cat# B8434, RRID:AB_476811). For visualization of primary antibodies, fluorescein-labelled anti-mouse immunoglobulin G (IgG; Thermo Fisher Scientific Cat# A-11001, RRID:AB_2534069), anti-rabbit IgG (Thermo Fisher Scientific Cat# A-21206, RRID:AB_2535792), or anti-goat IgG (Thermo Fisher Scientific Cat# A-11057, RRID:AB_2534104) were used (dilution 1:200). All cells were counterstained with Hoechst 33342.

Pictures were taken using an inverted fluorescence phase-contrast microscope (Keyence-3000). All immunohistochemical experiments were performed in triplicate.

### 2.15 Statistical analysis

Statistical analyses and graphics were created using GraphPad Prism (Version 6.01, GraphPad Software Inc., San Diego, CA, United States). All data are expressed as mean ± standard error of the mean (SEM). The Shapiro-Wilk test for normal distribution and Levene’s test of variance homogeneity were performed. If all variables analyzed met the assumption of normality, a *t*-test or one-way Analysis of Variance (ANOVA) was conducted to compare multiple groups. In case parameters turned out to be not normally distributed, a Mann-Whitney-U-test for comparison between two groups and one-way non-parametric ANOVA (Kruskal–Wallis test) for comparison between more than 2 groups were calculated and followed up by Dunn’s multiple comparison test. *p*-values that show statistical significance will be highlighted as followed: *p < 0.05, **p < 0.01, ***p < 0.001.

## 3 Results

### 3.1 Paclitaxel enters the central nervous system as well as dorsal root ganglia after intravenous application in mice

**FIGURE 1:**
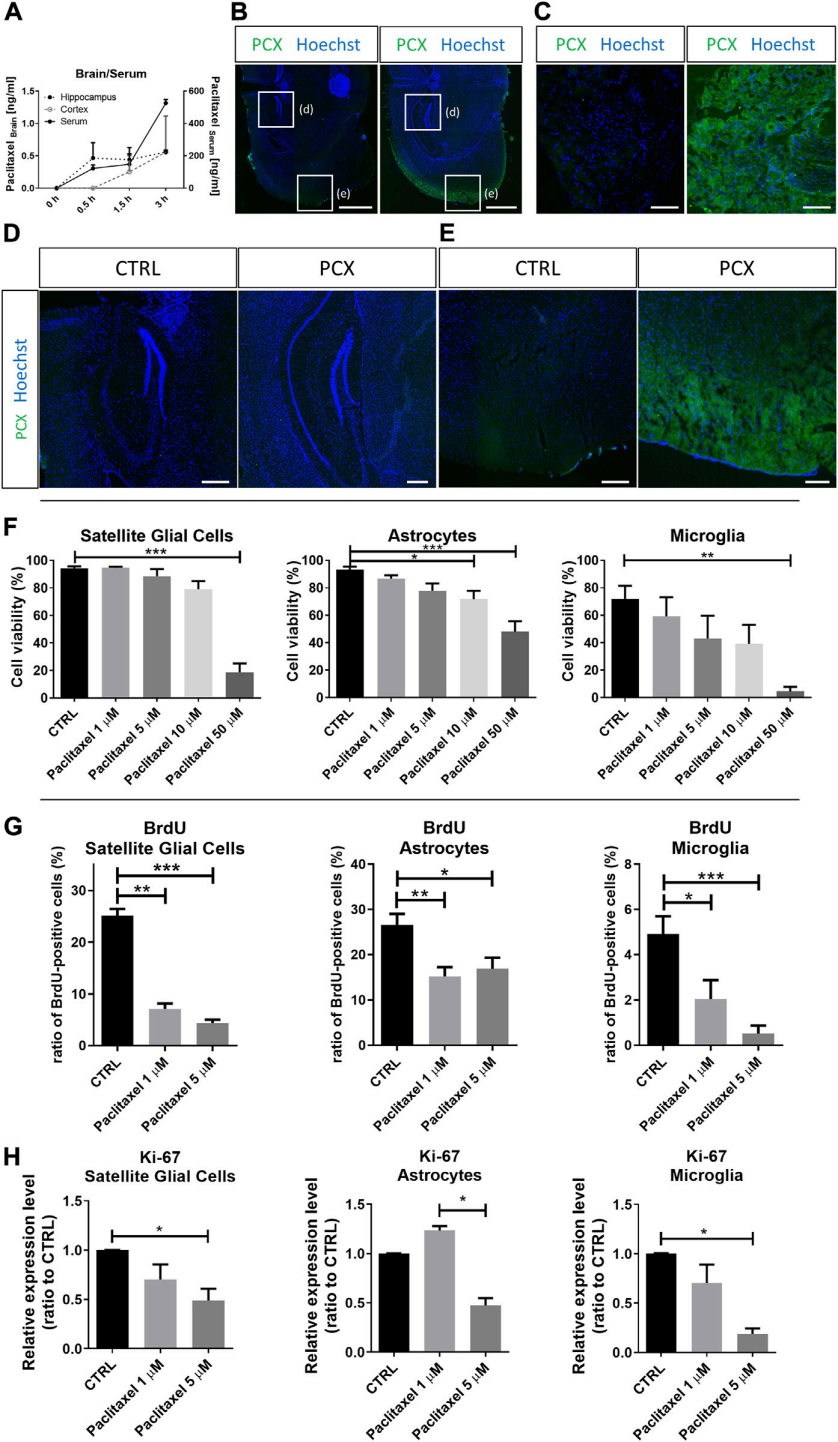
Efect of paclitaxel on survival and proliferation of glial cells. **(A)** 8-week-old mice were injected with 25 mg/kg paclitaxel (PCX) and paclitaxel amounts in serum (*n* (animals) = 3), cortex (*n* (animals) = 2), and hippocampus (*n* (animals) = 2) were assessed 0.5 h, 1.5 h, and 3 h after injection. The highest amounts of paclitaxel were detected after 3 h. Graphs depict mean ± SEM. **(B)** Paclitaxel immunohistochemistry in the mouse brain after 3 h of injection and control-treated mouse. Scale bar = 500 μm. **(C)** Paclitaxel immunohistochemistry in the mouse dorsal root ganglia (DRG) after 3 h of injection, compared to control mouse. Scale bar = 100 μm. **(D)** Paclitaxel immunohistochemistry in the hippocampal area after 3 h of injection, compared to control mouse. Scale bar = 100 μm. **(E)** Paclitaxel immunohistochemistry in the cortex area after 3 h of injection, compared to control mouse. Scale bar = 100 μm. **(F)** Cell viability of satellite glial cells (n (number of biological replicates) = 14), astrocytes (n (number of biological replicates) = 7) and microglia (n (number of biological replicates) = 5) after 24 h exposure to 1 μM, 5 μM, 10 μM, and 50 μM paclitaxel compared to unexposed (CTRL) cells. Graphs depict mean ± standard error of the mean (SEM). **(G)** Ratio of bromodeoxyuridine (BrdU)-positive cells of satellite glial cells (n (number of biological replicates) = 10), astrocytes (*n* (number of biological replicates) = 10) and microglia (*n* (number of biological replicates) = 10) after 24 h exposure to 1 μM and 5 μM paclitaxel compared to unexposed (CTRL) cells. Graphs depict mean ± SEM. **(H)** Relative expression level of Ki-67 in satellite glial cells, astrocytes, and microglia after 24 h exposure to 1 μM and 5 μM paclitaxel compared to unexposed (CTRL) cells (*n* (number of independent cell culture preparations) = 3). Graphs depict mean ± SEM. Statistical comparison was performed between all groups (Kruskal-Wallis test, Dunn’s multiple comparison test); *p < 0.05, **p < 0.01, ***p < 0.001.

We conducted spectrometric analyses over time to assess whether paclitaxel crosses the blood-brain barrier and accumulates in the CNS after intravenous application in mice. Indeed, paclitaxel started to accumulate in the hippocampus and cortex of the mouse brain already 30 min after intravenous injection (Figure 1A). The highest paclitaxel concentrations of 0.56 ng/ml were detected 3 h after injection in both cortex and hippocampus. Immunohistochemical stainings of brain slices confirmed the presence of paclitaxel with a higher signal in the cortex (Figures 1B,E) than in the hippocampus (Figures 1B,D). Moreover, immunohistochemical stainings revealed paclitaxel accumulation in DRG 3 h after injection (Figure 1C).

### 3.2 Paclitaxel dose-dependently impairs survival and reduces proliferation in astrocytes, microglia, and satellite glial cells *in vitro*

SGCs derived from DRG and astrocytes and microglia derived from the cortex of neonatal Wistar rats (P1-P3) were treated with different concentrations of paclitaxel *in vitro*. Cell viability assays showed that all 3 cell types reacted with a dose-dependent significant reduction of viable cells (Figure 1F & Supplementary Figure S1). The highest toxicity was observed when cells were treated with 50 µM paclitaxel, with a survival rate of 18.6% for SGCs (*p* < 0.001), 48.1% for astrocytes (*p* < 0.001), and only 4.5% for microglia (*p* < 0.01) compared to respective control. Of note, there was no significant cell death observed at lower doses of paclitaxel (1 μM and 5 µM) in any cell type. Therefore, we chose to use these lower concentrations for all subsequent experiments investigating the functional effects of paclitaxel.

Proliferating glial cells were incubated with BrdU, which is incorporated into dividing cells, allowing to calculate their proliferation rate. The proliferation of all glial cell types was negatively affected by paclitaxel treatment. Paclitaxel reduced the proliferation rate of SGCs from 25.14% to 7.07% at a concentration of 1 µM (*p* < 0.01), and to 4.3% at a concentration of 5 µM (*p* < 0.001). In astrocytes, the proliferation rate was reduced from 26.54% to 15.21% by 1 µM paclitaxel (*p* < 0.01), and to 16.91% by 5 µM paclitaxel (*p* < 0.05). The proliferation rate of microglia was reduced from 4.91% to 2.05% by 1 µM paclitaxel (*p* < 0.05), and to 0.52% by 5 µM paclitaxel (*p* < 0.001; Figure 1G).

Changes in BrdU incorporation were paralleled by the expression of Ki67 on the RNA level as an independent marker of cell proliferation. Exposure to 5 µM paclitaxel resulted in a 0.49-fold expression of Ki67 in SGC, 0.47-fold in astrocytes and 0.19-fold in microglia compared to control (for SGCs *p* < 0.05, microglia *p* < 0.05 compared to control; astrocytes *p* < 0.05 compared to the paclitaxel 1 µM-treated group; Figure 1H).

### 3.3 Activation of satellite glia cells by paclitaxel promotes a predominantly neurotoxic phenotype

**FIGURE 2:**
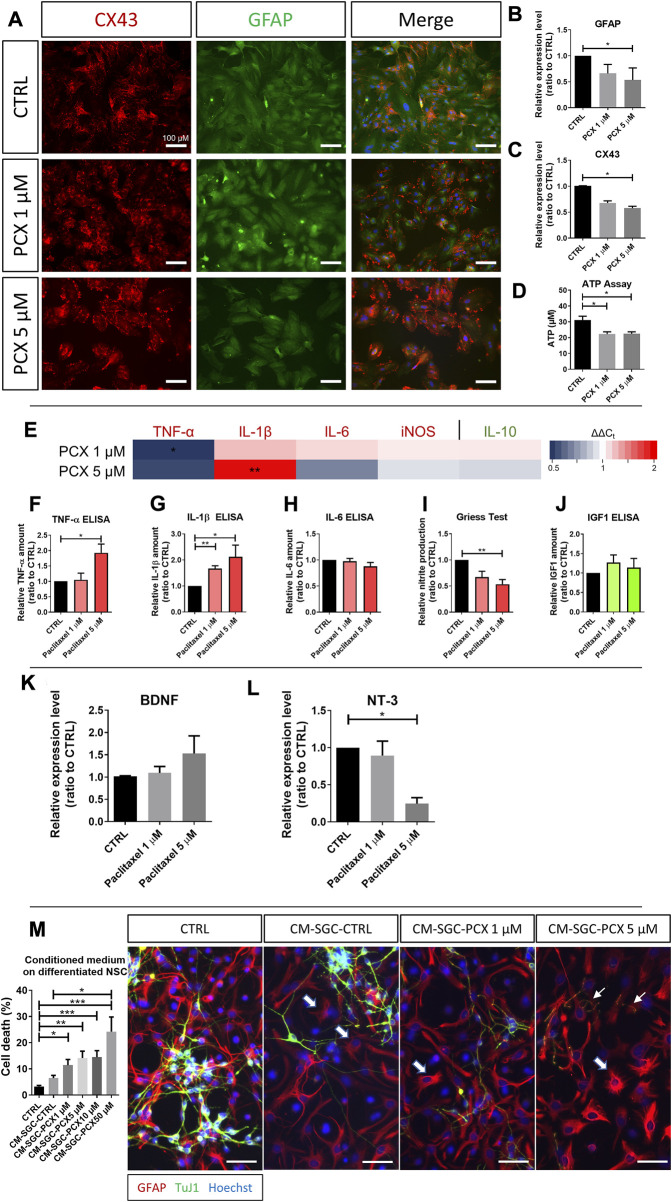
Effects of paclitaxel on satellite glial cells. **(A)** Immunofluorescence micrographs of Connexin 43 (CX43) and glial fibrillary acidic protein (GFAP) in satellite glial cells (SGC) after no treatment or exposure to 1 μM and 5 μM paclitaxel (PCX). **(B)** Relative expression level of GFAP in SGC after 1 μM and 5 μM paclitaxel exposure compared to control (n (number of independent cell culture preparations) = 3). **(C)** Relative expression level of CX43 in SGC after 1 μM and 5 μM paclitaxel exposure compared to control (n (number of independent cell culture preparations) = 3). **(D)** Released adenosine triphosphate (ATP) in μM after paclitaxel exposure to 1 μM and 5 μM compared to untreated (CTRL) cells (n (number of biological replicates) = 12). **(E)** Heatmap of pro-inflammatory markers, tumor necrosis factor-α (TNF-α), Interleukin-1 β (IL-1β), IL-6 and iNOS, and anti-inflammatory, IL-10, expression after 1 μM and 5 μM paclitaxel exposure depicted as 2^(−ΔΔCT)^. Measured relative amount of pro-inflammatory TNF-α **(F)** (n (number of independent cell culture preparations) = 3), IL-1β **(G)** (n (number of independent cell culture preparations) = 6), and IL-6 **(H)** (n (number of independent cell culture preparations) = 4) *via* enzyme-linked immunosorbent assay (ELISA). For an easier interpretation of the data, pro-inflammatory markers are depicted in red, anti-inflammatory markers are depicted in green. **(I)** Measured relative nitric oxide (NO) production assessed *via* Griess assay in 1 μM and 5 μM paclitaxel treated SGC compared to unexposed (CTRL) SGC (n (number of independent cell culture preparations) = 6). Measured relative amount of insulin-like growth factor 1 (IGF1) **(J)** *via* ELISA (*n* (number of independent cell culture preparations) = 4). **(K)** Relative expression level of brain-derived neurotrophic factor (BDNF) in SGC after 1 μM and 5 μM paclitaxel exposure compared to control (n (number of independent cell culture preparations) = 6). (L) Relative expression level of neurotrophin-3 (NT-3) in SGC after 1 μM and 5 μM paclitaxel exposure compared to control (*n* (number of independent cell culture preparations) = 3). **(M)** SGC were treated for 24 h with 1 μM, 5 μM, 10 μM, and 50 μM paclitaxel before medium was exchanged and cells were cultured for further 24 h. This conditioned medium (CM-SGC-CTRL or CM-SGC-PCX) was used in the following experiments. A neuron-glia co-culture was exposed to conditioned medium vs. control, and cell death was assessed (*n* (number of biological replicates) = 15). Further, immunohistochemistry of glial GFAP, Neuron-specific class III beta-tubulin (TuJ1), and Hoechst was performed on the co-culture after no exposure (CTRL) or exposure to conditioned medium. Immunocytochemical stainings revealed a loss of neurons upon exposure to CM-SGC-PCX, and the remaining neurons showed fragmentation and shortening of their neurites, indicating neurodegeneration (slim arrows). GFAP-positive astrocytes showed with a hypertrophic cell body and short thick and ramified processes (thick arrows) after exposure to CM-SGC-PCX. Scale bar = 100 μm. Graphs depict mean ± standard error of the mean (SEM). Statistical comparison was performed between all groups (Kruskal–Wallis test, Dunn’s multiple comparison test); *p < 0.05, **p < 0.01, ***p < 0.001.

To characterize our SGC culture, we conducted immunohistochemical stainings showing robust expression of GFAP and CX43, as described and discussed previously (Tongtako et al., 2017). As assessed by immunofluorescence, there was no visible change in CX43- or GFAP-protein expression in SGCs after paclitaxel exposure (Figure 2A). However, on the RNA level, paclitaxel at 5 µM decreased GFAP expression by 46% (*p* < 0.05), and CX43 by 42% (*p* < 0.05; Figures 2B,C) compared to control. In line with the decreased expression of CX43, extracellular ATP levels decreased upon exposure to paclitaxel compared to control (from 31.12 μM ATP in control to 22.29 μM ATP after 1 µM paclitaxel and 22.47 μM ATP after 5 µM paclitaxel exposure; *p* < 0.05; Figure 2D).

We next investigated the effects of paclitaxel on the immune competence of SGSc as measured by their ability to secrete chemokines and cytokines. The pro-inflammatory cytokine TNF-α was downregulated by 48% on mRNA level in SGCs after exposure to 1 µM paclitaxel (*p* < 0.05), whereas it was upregulated on the protein level as measured by ELISA in the supernatant of cultured SGCs after 5 µM paclitaxel exposure (1.93-fold compared to control, *p* < 0.05; Figures 2E,F). IL-1β as a second pro-inflammatory cytokine was upregulated 1.98-fold in SGCs on mRNA level (*p* < 0.01; paclitaxel 5 µM compared to control), as well as 1.8-fold on the protein level, after paclitaxel (*p* < 0.05; compared to control; Figures 2E,G). Expression of other pro-inflammatory markers IL-6 and nitric oxide synthase isoform (iNOS) remained unaffected (Figures 2E,H). In contrast, NO amounts decreased by 47% in the supernatant of SGCs treated with 5 µM paclitaxel as measured by Griess assay (*p* < 0.01; Figure 2I). However, levels of anti-inflammatory cytokine IGF-1 did not change in the presence of paclitaxel (Figures 2E,J).

Besides their involvement in the inflammatory response, glia cells are major suppliers of neurotrophic factors that foster the survival, growth, and differentiation of neurons. Brain-derived neurotrophic factor (BDNF) and neurotrophin-3 (NT-3) are the most prominent nerve growth factors released by glia. The expression of BDNF-RNA in SGCs was unaffected by paclitaxel (Figure 2K). However, NT-3 expression was dose-dependently decreased by 75% (5 µM paclitaxel, *p* < 0.05; Figure 2L).

To investigate the effects of paclitaxel-exposed SGCs on neurons *via* soluble factors, specifically their secretome, we first established a neuron-glia co-culture by targeted differentiation of primary NSC for 7 days. At this time point, robust young neurons, astrocytes, a few oligodendrocytes, and residual undifferentiated NSCs are present in the differentiating cultures (Vay et al., 2016; Vay et al., 2018). We used this co-culture model to compare the effects of the SGC secretome to the astrocyte secretome. After exposure to paclitaxel in various concentrations for 24 h, the medium was exchanged, and SGCs were cultured for an additional 24 h without paclitaxel before their conditioned medium containing the secretome (CM-SGC) was applied to the neuron-glia co-culture (Supplementary Figure S2). There was a significant paclitaxel-dose-dependent increase in cell death in co-cultures that received CM of paclitaxel-exposed SGC compared to control (*p* < 0.05 with CM-SGC-PCX1µM 11.41% cell death, *p* < 0.01 with CM-SGC-PCX5µM 14.14% cell death, *p* < 0.001 with CM-SGC-PCX10µM 14.44% cell death, *p* < 0.001 with CM-SGC-PCX50µM 24.24% cell death compared to control 3.26% cell death; Figure 2M). Additionally, immunocytochemical stainings revealed a loss of neurons upon exposure to CM-SGC-PCX, and the remaining neurons showed fragmentation and shortening of their neurites, indicating neurodegeneration (slim arrows; Figure 2M). Thus, data suggest that the secretome of SGCs exposed to paclitaxel exerts neurotoxic effects. Interestingly, concerning the morphology of GFAP-positive astrocytes, immunocytochemical stainings detected activated GFAP-positive astrocytes after treatment with the CM of paclitaxel-exposed SGCs, with a hypertrophic cell body and short thick and ramified processes (thick arrows; Figure 2M).

In summary, data suggest that SGCs are dose-dependently affected by paclitaxel, with reduced hemichannel activity, slightly enhanced pro-inflammatory activation, reduced neurotrophic and enhanced neurotoxic effects.

### 3.4 Astrocytes enhance the expression of neuroprotective markers and switch to an anti-inflammatory phenotype after paclitaxel treatment

**FIGURE 3:**
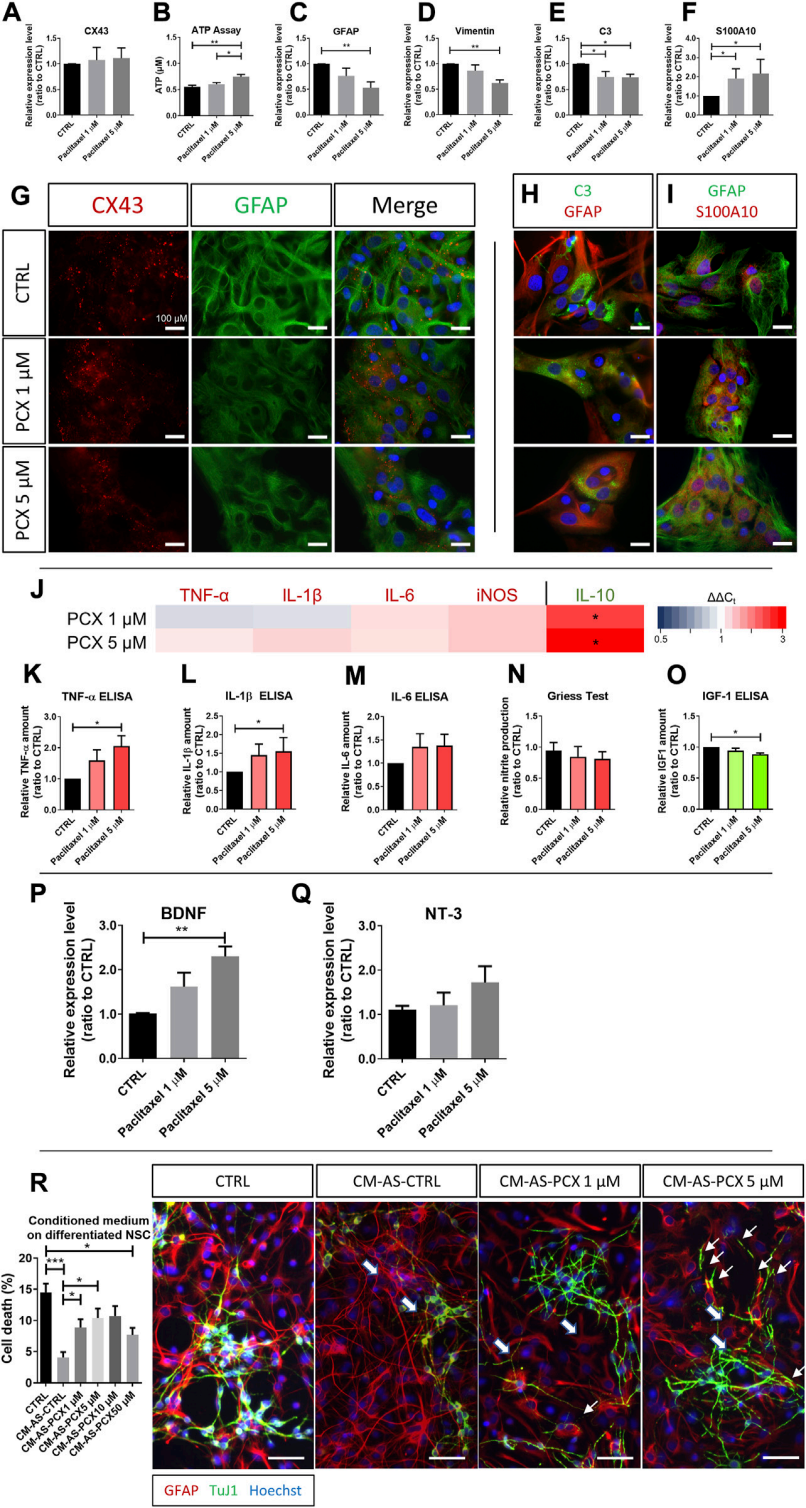
Effects of paclitaxel on astrocytes. **(A)** Relative expression level of Connexin 43 (CX43) in astrocytes after 1 μM and 5 μM paclitaxel exposure compared to control (*n* (number of independent cell culture preparations) = 6). **(B)** Released adenosine triphosphate (ATP) in μM after paclitaxel exposure with 1 μM and 5 μM compared to untreated (CTRL) cells (*n* (number of biological replicates) = 17). **(C)** Relative expression level of glial fibrillary acidic protein (GFAP) in astrocytes after 1 μM and 5 μM paclitaxel exposure compared to control (*n* (number of independent cell culture preparations) = 5). **(D)** Relative expression level of vimentin in astrocytes after 1 μM and 5 μM paclitaxel exposure compared to control (n (number of independent cell culture preparations) = 5). **(E)** Relative expression level of Complement 3 (C3) in astrocytes after 1 μM and 5 μM paclitaxel exposure compared to control. **(F)** Relative expression level of S100A10 in astrocytes after 1 μM and 5 μM paclitaxel exposure compared to control (*n* (number of independent cell culture preparations) = 4). **(G)** Immunofluorescence micrographs of CX43 and GFAP in astrocyte cultures after no exposure (CTRL) or exposure to 1 μM and 5 μM paclitaxel (PCX). **(H)** Immunofluorescence micrographs of C3 and GFAP in astrocyte cultures after no exposure (CTRL) or exposure to 1 μM and 5 μM paclitaxel (PCX). **(I)** Immunofluorescence micrographs of GFAP and S100A10 in astrocyte cultures after no exposure (CTRL) or exposure to 1 μM and 5 μM PCX. **(J)** Heatmap of pro-inflammatory markers, tumor necrosis factor-α (TNF-α), Interleukin-1 β (IL-1β), IL-6 and iNOS, and anti-inflammatory, IL-10, expression after 1 μM and 5 μM paclitaxel exposure depicted as 2^(−ΔΔCT)^. For an easier interpretation of the data, pro-inflammatory markers are depicted in red, anti-inflammatory markers are depicted in green. Measured relative amount of pro-inflammatory TNF-α **(K)**, IL-1β **(L)**, and IL-6 (m) *via* enzyme-linked immunosorbent assay (ELISA) (*n* (number of independent cell culture preparations) = 4). (*n*) Measured relative nitric oxide (NO) production assessed *via* Griess assay in 1 μM and 5 μM paclitaxel exposed astrocytes compared to unexposed (CTRL) astrocytes (*n* (number of independent cell culture preparations) = 4). **(O)** Measured relative amount of anti-inflammatory insulin-like growth factor 1 (IGF1) *via* ELISA (*n* (number of independent cell culture preparations) = 4). **(P)** Relative expression level of brain-derived neurotrophic factor (BDNF) in astrocytes after 1 μM and 5 μM paclitaxel exposure compared to control (*n* (number of independent cell culture preparations) = 6). **(Q)** Relative expression level of neurotrophin-3 (NT-3) in astrocytes after 1 μM and 5 μM paclitaxel exposure compared to control (*n* (number of independent cell culture preparations) = 6). **(R)** Astrocytes were exposed to 1 μM, 5 μM, 10 μM, and 50 μM paclitaxel for 24 h before medium was exchanged and cells were cultured for further 24 h. This conditioned medium was used in the following experiments. Neuron-glia co-cultures were treated with conditioned medium vs. control medium, and cell death was assessed (*n* (number of biological replicates) = 15). Further, immunohistochemistry of GFAP, Neuron-specific class III beta-tubulin (TuJ1), and Hoechst was performed on neuronal stem cells (NSC) after no exposure (CTRL) or exposure to conditioned medium. Immunocytochemical stainings revealed a loss of neurons upon exposure to CM-AS-PCX, and the remaining neurons showed fragmentation and shortening of their neurites, indicating neurodegeneration (slim arrows). GFAP-positive astrocytes showed with a hypertrophic cell body and short thick and ramified processes (thick arrows) after exposure to CM-AS-PCX. Scale bar = 100 μm. Graphs depict mean ± standard error of the mean (SEM). Statistical comparison was performed between all groups (Kruskal–Wallis test, Dunn’s multiple comparison test); *p < 0.05, **p < 0.01, ***p < 0.001.

In contrast to the findings on paclitaxel-induced CX43 expression in SGCs, CX43 expression in primary astrocytes was neither altered on the protein nor the mRNA level following exposure to paclitaxel (Figures 3A,G). Interestingly, ATP production dose-dependently increased upon paclitaxel exposure compared to control (paclitaxel 5 µM: *p* < 0.001; Figure 3B), suggesting an enhanced hemichannel activity that was not reflected by CX43 expression. Similarly to SGC, GFAP was downregulated on the mRNA level following paclitaxel exposure (e.g., by 46% after 5 µM paclitaxel treatment compared to control; *p* < 0.01; Figures 3C,G). In parallel, the downregulation of vimentin, a common activation marker for astrocytes was downregulated on the mRNA level of treated astrocytes by 40% compared to the control (paclitaxel 5 µM: *p* < 0.01; Figure 3D). To assess the effects on astrocyte phenotype, expression levels of complement component 3 (C3) as a marker for neurotoxic astrocytes, and S100A10 as a marker for neuroprotective astrocytes, were investigated on RNA level. While exposure to paclitaxel at 1 µM or 5 µM decreased C3 mRNA expression by 25% and 26% respectively (*p* < 0.05; Figures 3E,H), S100A10 expression was increased by paclitaxel exposure (1.91-fold after 1 μM, 2.17-fold after 5 µM paclitaxel; *p* < 0.05; Figures 3F,I), suggesting that paclitaxel led to the polarization of astrocytes toward a neuroprotective phenotype.

In primary astrocytes, paclitaxel did not affect the expression of pro-inflammatory cytokines (TNF-α, IL-1β, IL6) or iNOS on mRNA level (Figure 3J). However, translation of TNF-α and IL-1β was slightly enhanced, as measured by ELISA (increase of 2.05-fold and 1.55-fold at 5 µM paclitaxel compared to control; *p* < 0.05; Figures 3J,K). However, paclitaxel led to a robust dose-dependent upregulation of the anti-inflammatory cytokine IL-10 on mRNA level in astrocytes (2.49-fold after 1 μM, 3.06-fold after 5 µM paclitaxel, compared to control; *p* < 0.05; Figure 3J). At the same time, a second anti-inflammatory marker, IGF-1, was slightly decreased by 12% after exposure to 5 µM paclitaxel (*p* < 0.05, Figure 3O).

Equivalent to the SGCs in DRG of the PNS, astrocytes of the CNS constitute the main distributors of neurotrophic factors, expressing–among others–BDNF and NT-3. Paclitaxel exposure dose-dependently increased the expression of BDNF in astrocytes (*p* < 0.01; Figure 3P) while affecting NT-3 expression only by trend (Figure 3Q).

To investigate the effects of paclitaxel-exposed astrocytes on neurons, we employed the neuron-glia co-cultures derived from differentiating NSCs as detailed above. The secretome of naïve astrocytes exerted strong neuroprotective effects, as expected from their physiological role (CM-AS-control led to 4.1% cell death ratio, compared to control cultures with 14.5% cell death; *p* < 0.001; Figure 3R). This robust effect was partly preserved when astrocytes had previously been exposed to paclitaxel, but paclitaxel-exposed astrocytes did not support neurons as effectively as naïve astrocytes (*p* < 0.05 with CM-AS-PCX1µM 8.9% cell death, *p* < 0.05 with CM-AS-PCX5µM 10.4% cell death compared to CM-AS-CTRL 14.5% cell death; *p* < 0.001 with CM-AS-CTRL 14.5% cell death, *p* < 0.05 with CM-AS-PCX50µM 7.7% cell death compared to CTRL 4.1% cell death; Figure 3R). Moreover, staining for young neurons with neuron-specific class III beta-tubulin (TuJ1) showed some degradation with enhanced fragmentation of neurites after exposure to the secretome of paclitaxel-exposed astrocytes (small arrows) compared to control, suggesting some neurotoxic effects. Furthermore, fewer ramified GFAP-positive astrocytes were visible after exposure to the secretome of astrocytes exposed to paclitaxel (thick arrows; Figure 3R).

Overall, data suggest that astrocytes are affected by paclitaxel, showing slightly enhanced hemichannel activity and a higher expression of neuroprotective markers, while pro-inflammatory markers were reduced. This astrocyte activation phenotype lacked relevant pro-inflammatory functions but exerted neurotrophic effects, presumably by enhanced BDNF release.

### 3.5 Paclitaxel induces a pro-inflammatory, neurotoxic phenotype in microglia

**FIGURE 4:**
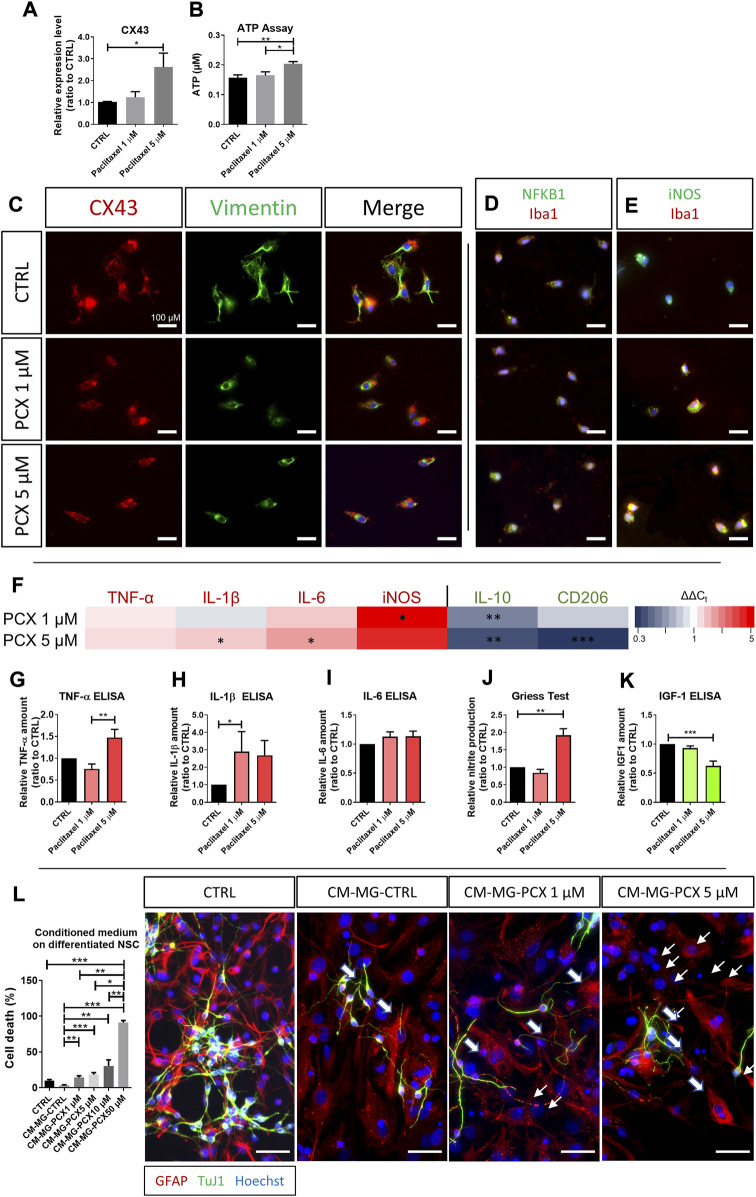
Effects of paclitaxel on microglia. **(A)** Relative expression level of Connexin (CX43) in microglia after 1 μM and 5 μM paclitaxel exposure compared to control (*n* (number of independent cell culture preparations) = 6). **(B)** Released adenosine triphosphate (ATP) in μM after paclitaxel exposure with 1 μM and 5 μM compared to unexposed (CTRL) cells (*n* (number of biological replicates) = 10). **(C)** Immunofluorescence micrographs of CX43 and vimentin in microglia after no exposure (CTRL) or exposure to 1 μM and 5 μM paclitaxel (PCX). **(D)** Immunofluorescence micrographs of nuclear factor kappa-light-chain-enhancer of activated B cells (NFκB1) and ionized calcium-binding adapter molecule 1 (Iba1) in microglia after no exposure or exposure to 1 μM and 5 μM PCX. **(E)** Immunofluorescence micrographs of iNOS and Iba1 in microglia after no exposure or exposure to 1 μM and 5 μM PCX. **(F)** Heatmap of pro-inflammatory markers, tumor necrosis factor-α (TNF-α), Interleukin-1 β (IL-1β), IL-6 and iNOS, and anti-inflammatory, IL-10 and cluster of differentiation 206 (CD206), expression after 1 μM and 5 μM paclitaxel treatment depicted as 2^(−ΔΔCT)^. For an easier interpretation of the data, pro-inflammatory markers are depicted in red, anti-inflammatory markers are depicted in green. Measured relative amount of pro-inflammatory TNF-α **(G)**, IL-1β **(H)**, and IL-6 **(I)** *via* enzyme-linked immunosorbent assay (ELISA) (n (number of independent cell culture preparations) = 4). **(J)** Measured relative nitric oxide (NO) production assessed *via* Griess assay in 1 μM and 5 μM paclitaxel exposed microglia compared to unexposed (CTRL) microglia (*n* (number of independent cell culture preparations) = 8). **(K)** Relative amount of anti-inflammatory insulin-like growth factor 1 (IGF1) *via* ELISA (*n* (number of independent cell culture preparations) = 5). **(L)** Microglia were exposed to 1 μM, 5 μM, 10 μM2, and 50 μM paclitaxel for 24 h before medium was exchanged and cells were cultured for further 24 h. Conditioned medium was used for the subsequent experiments. Neuron-glial co-cultures were treated with conditioned medium vs. control, and cell death was assessed (*n* (number of biological replicates) = 15). Further, immunohistochemistry of GFAP, neuron-specific class III beta-tubulin (TuJ1), and Hoechst was performed on neuronal stem cells (NSCs) after no exposure (CTRL) or exposure to conditioned medium. Immunocytochemical stainings revealed a loss of neurons upon exposure to CM-MG-PCX, and the remaining neurons showed fragmentation and shortening of their neurites, indicating neurodegeneration (slim arrows). GFAP-positive astrocytes showed with a hypertrophic cell body and short thick and ramified processes (thick arrows) after exposure to CM-MG-PCX. Scale bar = 100 μm. Graphs depict mean ± standard error of the mean (SEM). Statistical comparison was performed between all groups (Kruskal-Wallis test, Dunn’s multiple comparison test); *p < 0.05, **p < 0.01, ***p < 0.001.

Similar to the findings in astrocytes and in contrast to the findings in SGCs, CX43 expression and ATP-release dose-dependently increased in microglia cultures exposed to paclitaxel compared to control (*p* < 0.05; Figure 4A; ATP, *p* < 0.01; Figure 4B). Furthermore, upon paclitaxel exposure, microglia showed an increased expression of pro-inflammatory cytokines on the mRNA level (IL-1β, IL6; both *p* < 0.05; Figure 4F), as well as on protein level (TNF-α, *p* < 0.01; IL-1β, *p* < 0.05; Figures 4G,H). Moreover, the pro-inflammatory marker iNOS and the release of NO, increased after paclitaxel exposure (*p* < 0.05 and *p* < 0.01 respectively, Figures 4F,J). Upon paclitaxel exposure, microglia changed their morphology to adopt a spherical shape with fewer protrusions (Figure 4C), translocated NfkB from the cytoplasm into the nucleus (Figure 4D), and became immunoreactive for iNOS (Figure 4E), suggesting that paclitaxel induced a pro-inflammatory phenotype in microglia.

In line with this, anti-inflammatory markers dose-dependently decreased in primary microglia after exposure to paclitaxel. IL-10 mRNA expression was downregulated by 43% after microglia were exposed to 1 µM paclitaxel (*p* < 0.01) and by 61% after exposure to 5 µM paclitaxel (*p* < 0.01), compared to control (Figure 4F). The release of IGF-1 was downregulated by 38% as measured by ELISA (5 µM paclitaxel; *p* < 0.001; Figure 4K). Cluster of Differentiation 206 (CD206), another anti-inflammatory surface marker in microglia, was downregulated by 70% on mRNA level following exposure to 5 µM paclitaxel (*p* < 0.001; Figure 4F).

The secretome of paclitaxel-exposed microglia dose-dependently induced cell death in our neuron-glia co-culture, suggesting a neurotoxic effect. While in control co-cultures exposed to the secretome of naïve microglia, only 3.5% of cells were not viable, up to 91.5% of cells died upon exposure to the secretome of microglia exposed to 50 µM paclitaxel (*p* < 0.001; Figure 4L). Moreover, morphological analyses of those cultures revealed neuronal degeneration and fragmentation of neurites (thin arrows) and an increased number of hypertrophic GFAP-positive astrocytes (thick arrows; Figure 4M).

Overall, microglia exposed to paclitaxel increased their ATP release, predominantly adopting a pro-inflammatory microglial phenotype and their secretome dose-dependently mediated neurotoxic effects.

### 3.6 The secretome of astrocytes and satellite glial cells attenuates the pro-inflammatory microglia phenotype

**FIGURE 5:**
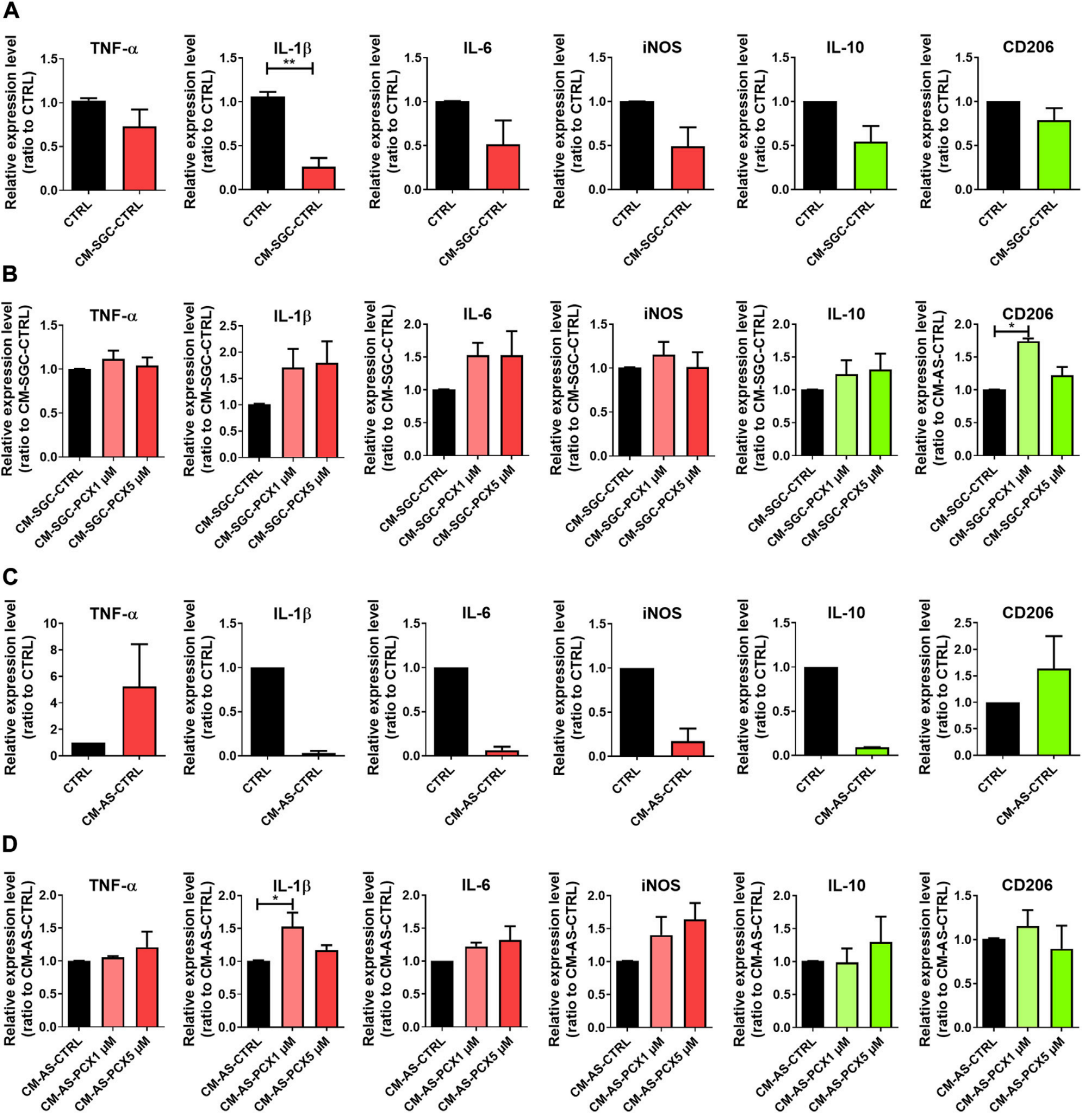
Effects of paclitaxel-exposed astrocytes and –satellite glial cells (SGCs) on microglia. SGC and astrocytes were exposed to 1 μM or 5 μM paclitaxel or received no paclitaxel for 24 h, before the medium was exchanged, and cells were cultured for further 24 h. For an easier interpretation of the data, pro-inflammatory markers are depicted in red, anti-inflammatory markers are depicted in green. **(A)** Microglia were exposed to the control secretome (CM-SGC-CTRL) of SGC and relative expression levels in ratio to control-exposed microglia (CTRL) of tumor necrosis factor-α (TNF-α), Interleukin-1 β (IL-1β), IL-6, iNOS, IL-10 and cluster of differenetiation 206 (CD206) were assessed (*n* (number of independent cell culture preparations) = 3). **(B)** Microglia were exposed to control secretome (CM-SGC-CTRL), 1 μM exposed secretome (CM-SGC-PCX1 μM) or 5 μM exposed secretome (CM-SGC-PCX5 μM) of SGC, and expression levels in ratio to CM-SGC-CTRL of TNF-α, IL-1β, IL-6, iNOS, IL-10 and CD206 were assessed (*n* (number of independent cell culture preparations) = 3). **(C)** Microglia cells were exposed to the control secretome (CM-AS-CTRL) of astrocytes, and relative expression levels in ratio to unexposed microglia (CTRL) of TNF-α, IL-1β, IL-6, iNOS, IL-10 and CD206 were assessed (*n* (number of independent cell culture preparations) = 2). **(D)** Microglia were exposed to the control secretome (CM-AS-CTRL), 1 μM exposed secretome (CM-AS-PCX1 μM), or 5 μM exposed secretome (CM-AS-PCX5 μM) astrocytes and expression levels in ratio to CM-AS-CTRL of TNF-α, IL-1β, IL-6, iNOS, IL-10 and CD206 were assessed (*n* (number of independent cell culture preparations) = 3). Graphs depict mean ± standard error of the mean (SEM). Statistical comparison was performed between all groups (Two groups: Mann-Whitney-U-test; multiple groups: Kruskal-Wallis test, Dunn’s multiple comparison test); *p < 0.05, **p < 0.01.

Glial cells intimately interact with each other. To assess the effects of paclitaxel on these interactions, microglia were exposed to the conditioned medium of astrocytes and SGCs that had in turn been exposed to paclitaxel. Interestingly, the secretome of both naïve SGCs and naïve astrocytes already reduced the expression of pro-inflammatory cytokines such as IL-1β from microglia (74% with CM-SGC-CTRL, *p* < 0.01; Figures 5A,C). Interestingly, we could not detect further differential effects on microglia activation after treatment with the secretome of paclitaxel-exposed SGCs or astrocytes (Figures 5B,D).

## 4 Discussion

Our study revealed that the chemotherapeutic drug paclitaxel enters the CNS and PNS and directly alters the function of resident glial cells, namely SGC of the PNS as well as astrocytes and microglia of the CNS. We also observed that paclitaxel differentially affects inter-glial communication and neuroinflammatory and neurotrophic activity of glia.

Our data demonstrating paclitaxel accumulation in the PNS corroborates previous studies describing an accumulation in the DRGs (Cavaletti et al., 2000). That is of immediate functional relevance since toxic substances cannot easily be cleared from the PNS as it is not connected to the lymphatic system (Kikuchi et al., 2006). Accordingly, a common side effect of paclitaxel is pain due to the accumulation of paclitaxel in sensory neurons (Argyriou et al., 2008; Carlson and Ocean, 2011). Paclitaxel disrupts microtubules in the mitotic spindle of dividing cells, but it may also disrupt microtubule-based transport in the axons of neurons, leading to neuronal injury and dysfunction. Hanani et al. proposed that the development of pain is due to neuronal injury that leads to the activation of SGCs *via* nitric oxide. SGC activation involves numerous processes, including greater coupling *via* gap junctions, increased sensitivity to ATP, upregulation of extracellular signal-regulated kinase (ERK), and increased release of pro-inflammatory cytokines, which altogether lead to neuronal excitation (spontaneous or evoked) of sensory neurons, resulting in pain (Warwick and Hanani, 2013). Furthermore, Watkins and colleagues (Watkins and Maier, 2003; Grace et al., 2014) proposed that microglia and astrocytes in the spinal cord participate in pain generation and maintenance and may serve as suitable targets for pain therapy. Based on our data, we firstly suggest that paclitaxel might have additional direct effects on SGCs, enhancing their pro-inflammatory activation and reducing their neurotrophic support by reduced expression of NT-3, probably both leading to the observed neurotoxic effects (cf. Figure 2). Hence, we assume that in paclitaxel-treated patients with neuropathic symptoms, the direct paclitaxel-induced impact on SGCs in the DRG might play an aggravating role in the induction and perpetuation of neurotoxicity.

An increase in SGC coupling has been observed after damage to sensory neurons and was proposed to be part of SGC activation as mentioned above (Cherkas et al., 2004; Dublin and Hanani, 2007; M. Hanani et al., 2002). Of note, our results revealed that under the influence of paclitaxel, CX43 expression and ATP release were significantly reduced, contradicting the current literature (cf. Figure 2) (Kim et al., 2016; Komiya et al., 2018). Neurons themselves can release ATP at their synapses (Fields and Stevens, 2000; Hamilton et al., 2008), along the axon, and at their cell body (X. Zhang et al., 2007). Neuronal ATP release fosters ATP excretion of SGCs that, in turn, release inflammatory cytokines IL-1β and TNF-α. This feedback loop strengthens neuronal excitability (Takeda et al., 2009; X. Zhang et al., 2007). Therefore, we speculate that SGCs function and expression profile might also be influenced by this neuronal-glial interaction, which is a limiting factor of our mono-SGC culture.

Similar to the investigations of Huehnchen et al., our study confirmed that paclitaxel can cross the blood-brain barrier and accumulates in the mammalian cortex and hippocampus. Interestingly, Gibson et al. recently suggested chronic microglia activation as a primary mechanism mediating oligodendrocyte depletion and astrocyte activation in the context of methotrexate-induced cognitive impairment (Gibson et al., 2019). In line with these findings, our data suggest a primary effect of paclitaxel on the activation of microglia and astrocytes and, thus, a possible new mechanism for chemotherapy-induced cognitive impairment.

In the past, two distinct phenotypes of activated astrocytes have been described, the A1 and A2, that can be identified *via* the expression of complement 3 (C3), complement factor B (CFB), and MX1S, expressed on A1 astrocytes, and S100A10, expressed on A2 astrocytes (Liddelow et al., 2017; Fujita et al., 2018). Activated microglia supposedly are able to induce the A1 type in astrocytes *via* IL-1α, TNF, and complement component 1q (C1q) cytokines (Liddelow et al., 2017). In turn, A1 astrocytes express pro-inflammatory cytokines and possibly neurotoxins, leading to the death of neurons and oligodendrocytes. A2 astrocytes, on the other hand, promote the expression of anti-inflammatory cytokines, e.g., TGF-β, which plays a role in synaptogenesis and neuroprotection (Xu et al., 2018). S100A10 is implicated in positive effects on cell proliferation, membrane repair, and cell survival (Liddelow et al., 2017).

Recently, it became apparent that there is not a strict polarization between A1 and A2 astrocytes, and that none of the marker genes of A1 or A2 are strictly toxic or protective (Escartin et al., 2021). This is in line with previous findings in microglia, where an initially proposed M1/M2 dichotomy was later withdrawn in favor of a more dynamic model (Vay et al., 2018). Across various neurological disorders, astrocytes present a diversity of phenotypes. Although A1 and A2 only describe two extremes on a continuum, this approximate categorization helps understand pathophysiological processes and should not be completely discarded. The current study suggests a paclitaxel-induced neuroprotective astrocyte phenotype consistent with predominantly neurotrophic and anti-inflammatory properties (cf. Figure 3). In various neuropathological processes, e.g., after ischemic stroke, in tumor-associated astrocytes, and after methotrexate-treatment, the astrocytic activation state is strongly regulated by microglia (Liddelow et al., 2017; Gibson et al., 2019; Henrik Heiland et al., 2019). Being the first to describe a switch of astrocytes to an anti-inflammatory phenotype could provide an an innovative potential therapeutic target.

Increased microglial IL-6 and TNF- α expression has been described in the spinal cord of mouse and rat models after paclitaxel exposure (Ha et al., 2019; Wu et al., 2019). In line with these studies, we show for the first time that paclitaxel directly leads to an activation of microglia releasing pro-inflammatory cytokines and ATP, with a loss of anti-inflammatory properties inducing predominantly neurotoxic effects (cf. Figure 4). Especially the release of TNF-α by microglia has been linked to neurotoxicity (Taylor et al., 2005; Kaushal and Schlichter, 2008). While ATP release can have neuroprotective effects, it was also described to directly induce neuronal and glial cell death (Amadio et al., 2002; Ryu et al., 2002). ATP itself can activate microglia and astrocytes in a positive feedback loop, thus leading to a prolonged activation state (Xin Zhang et al., 2014).

SGCs might be viewed as the counterpart to astrocytes in the PNS. SGCs and astrocytes are both homeostatic glia; however, in the past, astrocytes have been more extensively studied than SGCs. The main difference between these two glial cell types is that astrocytes are more diverse, e.g., in activation states, and have increased functional capabilities (Hanani and Verkhratsky, 2021). However, in our study, all three glial cells reacted differently to exposure to paclitaxel. While astrocytes in the CNS seem to be the population that sustains an anti-inflammatory profile, microglia and SGC, to a lower extent, responded with adverse detrimental effects, a pro-inflammatory profile and a negative impact on the survival of neurons.

Although the results provided are novel and, in detail, describe the phenotype and inflammatory states of glial cells after exposure to paclitaxel, this study has several limitations. So far, all results on glial phenotypes were raised from *in vitro* experiments. We demonstrated a dose-dependent effect of paclitaxel on primary cell cultures. However, time dependency of treatment was not evaluated. Concerning the fact that patients treated with paclitaxel not only suffer from tumour disease, which might influence the function of the immune system but also are treated with paclitaxel repetitively over a long period of time, these treatment effects are of interest for further studies. Moreover, we assume that the crosstalk between the different glial types and glia- neuronal cells might influence the actual extent of glial activation *in vivo*. Above that, *in vitro* glial cells might present with a different phenotype due to the preparation procedure, that additionally might differ depending on the time in culture conditions. Furthermore, we cannot exclude sex-specific differences, as our primary cultures were prepared from male and female animals. In sum, translation into *in vivo* is the next challenging step to further investigate and support our findings.

## Conclusion

Interestingly, the results of our study confirmed that all three types of glia had different and partly opposing roles in intercellular interaction, highlighting the complexity of pathophysiological glial processes. These multidimensional processes offer the potential to develop novel therapeutic approaches. Shedding light on the glial response to paclitaxel, our data contribute to the understanding of potential mechanistic pathways of paclitaxel-induced CNS and PNS dysfunction.

## Data Availability

The original contributions presented in the study are included in the article/Supplementary Material, further inquiries can be directed to the corresponding author.

## Glossary

ANOVA: Analysis of variance
ATP: Adenosine triphosphate
BDNF: Brain-derived neurotrophic factor
BrdU: Bromodeoxyuridine
C1q: complement component 1q
C3: Complement C3
CD206: Cluster of Differentiation 206
CFB: Complement factor B
CIPN: Chemotherapy-induced peripheral neuropathy
CM: Conditioned medium
CNS: Central nervous system
CT: Threshold cycle
CX43: Connexin 43
DMEM: Dulbecco’s essential medium
DNA: Deoxyribonucleic acid
DRG: Dorsal root ganglia
EDTA: Ethylenediaminetetraacetic acid
ELISA: Enzyme-linked immunosorbent assay
ERK: Extracellular signal-regulated kinase
FBS: Fetal bovine serum
FGF-2: Fibroblast growth factor
GFAP: Glial fibrillary acidic protein
Iba1: Ionized calcium-binding adapter molecule 1
IGF1: Insulin-like growth factor 1
IL: Interleukin
iNOS: Nitric oxide synthase isoform
LC-MS/S: Liquid chromatography– tandem mass spectrometry
NFκB: Nuclear Factor kappa-light-chain-enhancer of activated B cells
NO: Nitric oxide
NSCs: Neuronal stem cells
NT-3: Neurotrophin-3
PBS: Phosphate-buffered saline
PCX: Paclitaxel
PFA: Paraformaldehyde
PNS: Peripheral nervous system
RNA: Ribonucleic acid
RPL13a: 60S ribosomal protein L13a
RT-qPCR: Real-time quantitative polymerase chain reaction
S100A10: S100 calcium-binding protein A10
SEM: Standard error of the mean
SGCs: Satellite glial cells
TNF-α: Tumor necrosis factor-α
TuJ1: Neuron-specific class III beta-tubulin
