# Supplementary figures and images for: Glia from the central and peripheral nervous system are differentially affected by paclitaxel chemotherapy via modulating their neuroinflammatory and neuroregenerative properties
Source: Front Pharmacol. 2022 Nov 2;13:1038285. doi: 10.3389/fphar.2022.1038285 (PMC9666700; doi:10.3389/fphar.2022.1038285)

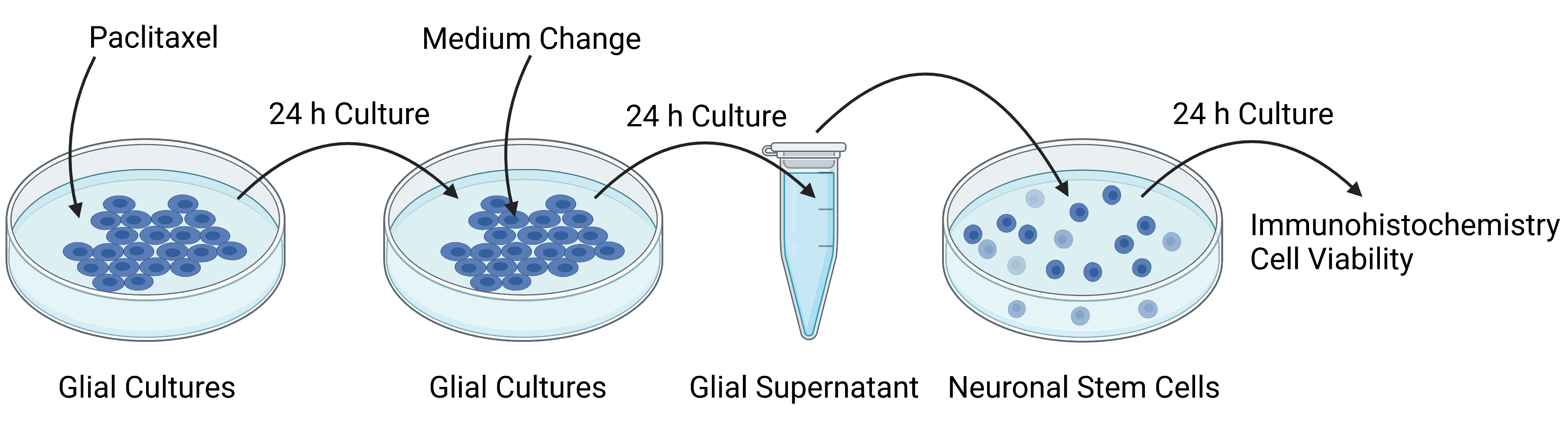

Supplement: Supplementary file 3 [file Image2.png]

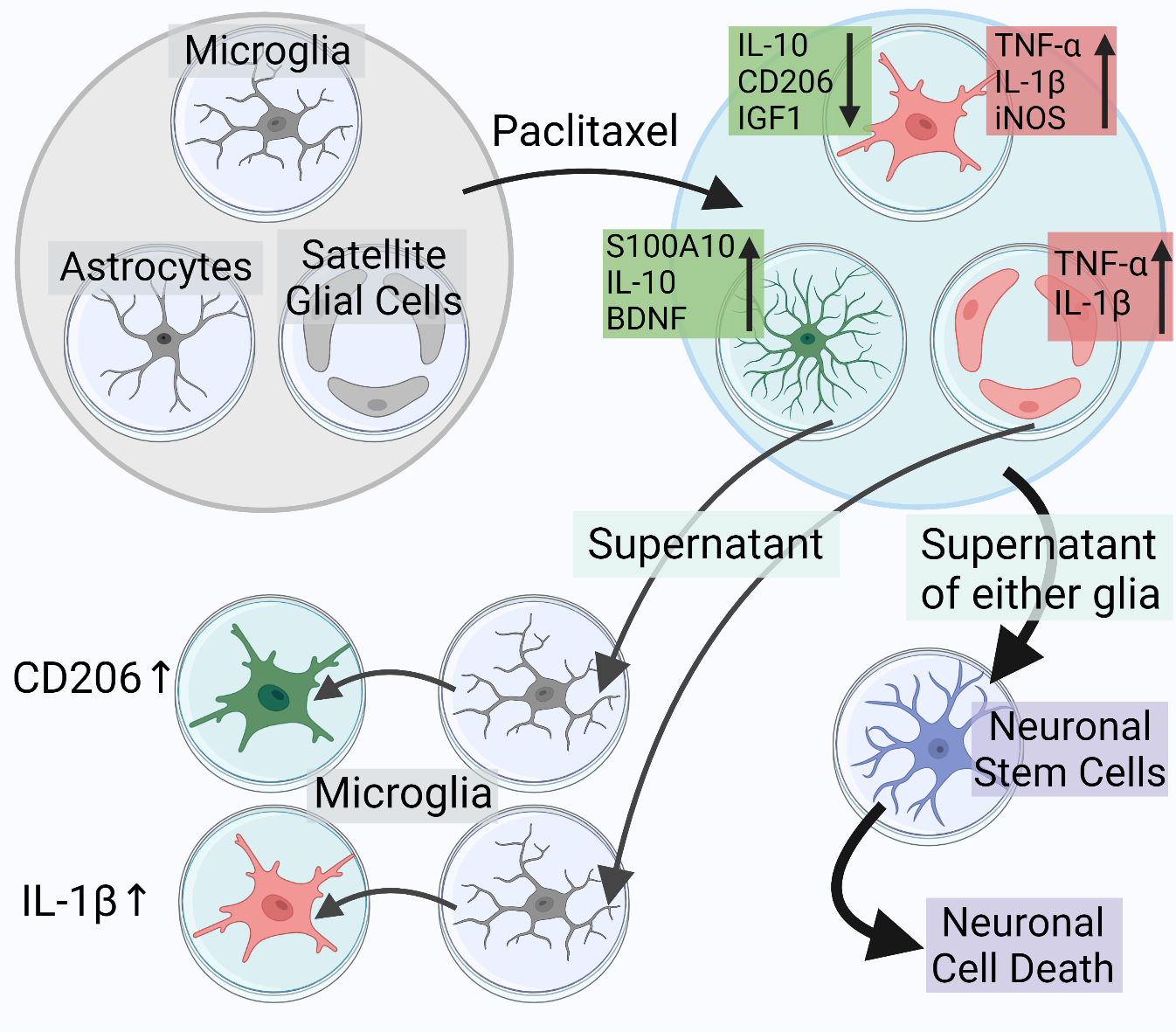

Supplement: Supplementary file 4 [file Image1.png]
